# Nanosystems Applied to HIV Infection: Prevention and Treatments

**DOI:** 10.3390/ijms21228647

**Published:** 2020-11-17

**Authors:** Micaela A. Macchione, Dariana Aristizabal Bedoya, Francisco N. Figueroa, María Ángeles Muñoz-Fernández, Miriam C. Strumia

**Affiliations:** 1 Departamento de Química Orgánica, Facultad de Ciencias Químicas, Universidad Nacional de Córdoba, Av. Haya de la Torre y Av. Medina Allende, Córdoba X5000HUA, Argentina; micaela.macchione@unc.edu.ar (M.A.M.); dariana.aristizabal@unc.edu.ar (D.A.B.); ffigueroa@unc.edu.ar (F.N.F.); 2 Instituto Académico Pedagógico de Ciencias Humanas, Universidad Nacional de Villa María, Arturo Jauretche 1555, Villa María, Córdoba X5220XAO, Argentina; 3 Instituto de Investigación y Desarrollo en Ingeniería de Procesos y Química Aplicada (IPQA), CONICET, Av. Velez Sárfield 1611, Córdoba X5000HUA, Argentina; 4 Immunology Section, Laboratorio InmunoBiología Molecular, Instituto Investigación Sanitaria Gregorio Marañón (IiSGM), Hospital General Universitario Gregorio Marañón (HGUGM), Spanish HIV HGM BioBank, C/Dr. Esquerdo 46, 28007 Madrid, Spain; mmunoz.hgugm@gmail.com; 5 Plataforma de Laboratorio, Hospital General Universitario Gregorio Marañón, 28007 Madrid, Spain; 6 Networking Research Center on Bioengineering, Biomaterials and Nanomedicine (CIBER-BBN), 28007 Madrid, Spain

**Keywords:** nanosystems, HIV infection, prevention, treatment, nanotechnology

## Abstract

Sexually-transmitted infections (STIs) are a global health concern worldwide as they cause acute diseases, infertility, and significant mortality. Among the bacterial, viral, and parasitic pathogens that can be sexually transmitted, human immunodeficiency virus (HIV) has caused one of the most important pandemic diseases, which is acquired immune deficiency syndrome (AIDS). 32.7 million people have died from AIDS-related illnesses since the start of the epidemic. Moreover, in 2019, 38 million people were living with HIV worldwide. The need to deal with this viral infection becomes more obvious, because it represents not only a problem for public health, but also a substantial economic problem. In this context, it is necessary to focus efforts on developing methods for prevention, detection and treatment of HIV infections that significantly reduce the number of newly infected people and provide a better quality of life for patients. For several decades, biomedical research has been developed allowing quick solutions through the contribution of effective tools. One of them is the use of polymers as vehicles, drug carrier agents, or as macromolecular prodrugs. Moreover, nanosystems (NSs) play an especially important role in the diagnosis, prevention, and therapy against HIV infection. The purpose of this work is to review recent research into diverse NSs as potential candidates for prevention and treatment of HIV infection. Firstly, this review highlights the advantages of using nanosized structures for these medical applications. Furthermore, we provide an overview of different types of NSs used for preventing or combating HIV infection. Then, we briefly evaluate the most recent developments associated with prevention and treatment alternatives. Additionally, the implications of using different NSs are also addressed.

## 1. Introduction

Sexually-transmitted infections (STIs) such as human immunodeficiency virus (HIV) infection are a global health concern worldwide as they cause acute diseases, infertility, and significant mortality. After the first patients were identified with acquired immune deficiency syndrome (AIDS) in 1981, and the association between HIV infection and AIDS that was realized some years later [1], HIV/AIDS became a global pandemic with huge health, social, and economical consequences. The Joint United Nations Program on HIV/AIDS (UNAIDS) estimates that approximately 38 million people were living with HIV worldwide in 2019, and 32.7 million people have died from AIDS-related illnesses since the start of the epidemic [2]. These alarming statistics have caused tremendous social and economic damage worldwide.

Two types of HIV are known to cause AIDS: HIV-1 and HIV-2. The former extends worldwide whereas the latter is present mainly in Western Africa and communities in Europe with socioeconomic links to West Africa. HIV-1 and HIV-2 share many similarities including their basic gene arrangement, intracellular replication pathways, modes of transmission, and clinical consequences. However, HIV-2 is characterized by lower transmissibility and a slower progression to AIDS [3,4].

Sexual transmission certainly remains the predominant mode of transmission [5]. Thanks to targeted prevention programs, new HIV infections have been reduced by 40% since the peak of the pandemic in 1998 [2]. In 2019, around 1.7 million people were newly infected with HIV, compared to 2.8 million people in 1998.

The global HIV epidemic is not homogeneously distributed. On the contrary, it is disproportionately concentrated in sub-Saharan Africa, where, in 2017, 75% of deaths and 65% of new infections occurred, and where 71% of people living with HIV resided [6,7].

In sub-Saharan Africa, women are more vulnerable than men to contracting HIV because of the higher biological susceptibility of women to HIV and sociodemographic factors such as gender-based violence, age discrepancy in relationships, and limited access to education [8]. Although an effective protective behavior to reduce HIV transmission is the use of male condoms, women in heterosexual relations require negotiation or consent of their male partners for their use [9]. Therefore, statistics indicate that every week, around 5500 young women aged 15–24 years become infected with HIV in this region. Thus, young women are twice as likely to be living with HIV as men [2].

People living with HIV are at increased risk of developing other infections such as tuberculosis, hepatitis B, and hepatitis C. During 2020 and with the quick spread of SARS-CoV-2 around the world, HIV/SARS-CoV-2 co-infection was expected to become a common phenomenon in countries with a high HIV prevalence [10]. In this context, it remains unclear whether people living with HIV are at increased risk of severe acute respiratory syndrome coronavirus manifestation. Therefore, in the lack of conclusive studies on HIV/SARS-CoV-2 co-infection, more research is needed to describe clinical manifestations of severe acute respiratory syndrome coronavirus among HIV patients and to determine optimal treatments [10,11,12].

Taking into account the high amount of people infected annually, it is evident that more efforts are required to deal with HIV viral infection. Therefore, the research community still has to face the development of advanced methods for prevention, detection, and treatment of HIV infections that significantly reduce the number of newly infected people and provide a better quality of life for patients.

Polymers have emerged as effective tools for developing strategies of diagnosis, prevention, and treatment of HIV infection. In particular, they have found application as drug carrier agents of antiviral drugs, development of microbicides, vaccines, gene therapy, among others.

Recent progress in the development of sub-micrometer systems opens the new prospective of introducing several nanoformulated structures to deal with this viral infection.

This review aims to summarize recent research into diverse nanosystems (NSs) as potential candidates for the prevention and treatment of HIV infection. Although we consider that the different research efforts made to improve diagnostic systems are valuable as well, this matter will not be addressed in this review. First, we provide an overview of characteristics and advantages of different types of polymers and nanosized structures used for HIV infection. Then, we briefly evaluate the most recent developments associated with prevention and treatment alternatives for HIV infection. The implications of using different NSs are also addressed.

### 1.1. Generalities of Different Types of Polymeric Systems

For several decades, biomedical research has developed quick solutions by providing effective tools for curing or mitigating HIV infection. One of them is the use of polymers as drug carrier agents or as macromolecular prodrugs. Biocompatible polymeric drug-containing systems have been found to exhibit lower toxicity, increase delivery to a specific site, improve drug stability and solubility, and display superior pharmacokinetics compared to non-polymeric drugs [13]. The concept of polymeric drug anchoring has been successfully applied to a variety of drugs and other hybrid systems, for example platinum, ferrocene, methotrexate, curcumin and in numerous therapies, such as cancer, osteoarthritis, viral diseases, etc. It was demonstrated through in vivo studies that conjugated systems based on covalently incorporated drug and polymeric carrier notably improve the half-life of the drug, confer an optimal sustained release profile, and reduce toxicity [14].

There are several types of polymeric systems that are used as drug carriers. These can be mainly divided into two types: natural or synthetic polymers. Some main structural characteristics are given below.

#### 1.1.1. Natural Polymers

They are polymers of natural origin with the advantage of being biocompatible, biodegradable, and having functional groups in their chemical structure that can be used to bind a drug or target molecule. The most used are polypeptides, polysaccharides, and glycoproteins [15]. Unfortunately, they have some deficiencies such as their rapid degradability in biological systems and poor mechanical resistance properties. Characteristic examples are starch and gelatin, which rapidly disintegrate and are removed due to their many biodegradable bonds, or polypeptides that are rapidly metabolized by proteolysis in most routes of administration [16]. In addition, the hydrophilicity of natural polymers gives them very low penetration into biomembranes. Due to these drawbacks, in most cases it is necessary to carry out chemical modifications for giving them suitable properties for use as vehicles in drug transport. Some of these modifications consist of crosslinking, grafting, or derivatization reactions of the functional groups present in their chemical structure (esterification, etherification, methylation, among others). There are some interesting examples which used natural polymers in HIV formulations. As example, numerous microbicides were scheduled to enter phase III clinical trials using carrageenan [15], cellulose sulphate [17], and dextrin-2-sulphate [18].

#### 1.1.2. Synthetic Polymers

The unquestionable advantage of synthetic polymers is their great versatility and chemical and mechanical stability. Different polymers, such as poly(ethylene glycol) (PEG), N-(2-hydroxypropyl)-methacrylamide (HPMA), poly(ethyleneimine) (PEI), poly(amidoamine) (PAA), poly(vinyl alcohol) (PVA), and poly(aspartamides) copolymers, have been synthesized and extensively used as vehicles for drug delivery.

Several antiviral drug carriers under investigations are based on the use of synthetic polymers. Antiviral drugs are used to treat viral infections, such as HIV, herpes, hepatitis B and C, herpes zoster, influenza A and B, and Ebola. However, knowing the contraindications of the direct application of these drugs, conjugated systems based on drug and polymeric carrier are used as an attractive strategy with better properties. Gunaseelan et al. developed a polymer conjugated of PEG with saquinavir that is an inhibitor of HIV-1 protease [19]. The drug was covalently attached to the polymer through an ester linkage with the amino group of cysteine, capable of being subsequently hydrolyzed. These systems were studied as anti-HIV-1 in MT-2 cells, giving better cell uptake as well as greater water solubility, plasma half-life, and antiviral activity in relation to the isolated drug. Another example is the work of Hiramath et al. in which polymer-drug conjugates of acyclovir were prepared and drug release at different pHs (1.2, 5.5, 6.8, and 7.4) was studied. The stability of the system and an increase in the duration of the acyclovir activity were observed, along with longer retention time in plasma and greater bioavailability [20]. Another example is the work of Vlieghe et al. which reported the incorporation of azidothymidine to k-carrageenan through the ester bond [21].

### 1.2. Generalities of Different Types of Nanosystems

Advances in nanoscience have demonstrated that NSs play a significant role in the prevention and therapy against HIV. The use of nanoformulations leads to sustained release of drugs and an increase in stable plasma concentration within the therapeutic range with reduced dose and frequency of administration [22,23]. In addition, the size on the nanometer scale allows NSs to enter hard-to-reach regions, such as the brain, providing protection for therapeutic agents while being administered in damaged areas. Moreover, NSs offer the versatility of being able to be functionalized with specific ligands and the advantage of being present mainly in the biological site of interest reducing the damage of other tissues. Nanoformulations for the prophylaxis and cure of HIV infection have been developed using a single or multi-drug, and to be administered intramuscularly, subcutaneously, or topically [22,23].

Some NSs used in HIV are mentioned below.

#### 1.2.1. Liposomes

Liposomes are lipid vesicles of variable size and structural complexity within which a variety of polar, apolar, and amphipathic drugs can be encapsulated. They are rapidly absorbed by phagocytic cells, such as the liver, spleen, and the reticuloendothelial system. Therefore, their application is inefficient in therapies such as cancer. This problem was solved by including a layer of a hydrophilic polymer, such as PEG, on its surface. However, the coating of liposomes by hydrophilic polymers is not always advantageous, since some of them are not degraded by mammalian enzymes and, consequently, the polymers can accumulate and cause long-term effects [24]. In other cases, they can hinder drug release and interaction with the cell membrane at the specific site where they should act, leading to a reduced therapeutic efficacy. Another disadvantage found in liposomes, both coated and uncoated, is that adverse effects at the respiratory and hemodynamic levels have been observed in the treatment of patients, causing hypersensitivity reactions.

#### 1.2.2. Polymeric Micelles

They are nanosized, self-assembled, supramolecular, colloidal, spherically-shaped systems with a hydrophobic core and a hydrophilic layer formed by aggregation [25]. The properties of micelles depend on the balance between the hydrophobic core and the hydrophilic layer, which give them their shape and size. The hydrophilic block produces biocompatibility whereas the hydrophobic block is used to encapsulate drugs in a wide variety of structures, lipophilicity, and charges. These advantages give micelles considerable applications as drug carriers. Block polymers that are frequently studied include PEG-poly(amino acids), PEG-poly(d, l-lactide) (PEG-PLA), PEG-poly(ε-caprolactone) (PEG-PCL), PEG-distearoylphosphatidylethanolamine (PEG-DSPE), and PEG-poly(propyl oxide)-PEG (PEG-PPO-PEG, Pluronics) [26].

#### 1.2.3. Dendrimers

They are hyperbranched and hyperfunctionalized three-dimensional synthetic macromolecules that offer multivalent interactions [27]. As they increase in generation, their shape changes from oval to spherical while their size remains within the nanoscale. They are characterized by having an easily controllable structure, a very low polydispersity index, and a large number of peripheral functional groups [28,29]. These groups can be used to bind a target molecule or a drug and manage solubility and stability in different biological media and pHs. Dendrimers are synthesized through an iterative method that can be convergent or divergent. This allows generational growth with the presence of internal cavities that can be used to encapsulate a wide variety of nanoparticles (NPs) or molecules such as drugs, imaging agents, and dyes.

In general, viruses adhere to host cells and enter them through multivalent interactions between viral ligands and receptors on the cell membrane. Thus, the use of monovalent drugs to inhibit viral entry and subsequent spread is not very effective due to the strong multivalent virus–cell interactions [30]. Consequently, there is a need to develop antiviral agents based on multivalent interactions to effectively protect virus particles and inhibit virus–cell interactions, preventing entry and subsequent infection [31]. However, it is challenging to maintain the host cell viability while targeting the virus.

#### 1.2.4. Nanogels

A nanogel (NG) is a three-dimensional crosslinked polymeric matrix that has nanometric sizes in its three dimensions. They have wide applications in nanomedicine given their high water retention capacity, their nanoscale size, and the versatility to be synthesized by using biocompatible polymers. Drug molecules encapsulated in NGs exhibit high stability, and furthermore, the crosslinking degree can be regulated to control drug encapsulation and release [32,33]. NGs can be synthesized by applying different methodologies, among which the most widely used are controlled and uncontrolled radical polymerization and “click” chemistry.

Additionally, NGs can be designed to shrink or swell in response to external stimuli to give ‘‘stimuli-responsive nanogels’’, pH, temperature, enzymes, light, and redox potential being the most important stimuli [34]. Among NGs, those which exhibit stimuli-responsiveness are considered highly advantageous because they can achieve a controlled release in the site of interest [35,36].

#### 1.2.5. Nanoparticles and Hybrid Systems

In the last two decades, the use of inorganic NPs such as magnetic, gold, mesoporous silica, quantum dots have gained a great interest in different fields. However, it is unusual that they can be used alone due to their stability problems. Thereby, it is necessary to functionalize their surface through different ways, organic compounds or polymeric materials generally being used for this purpose. The advantages of biomedical applications using inorganic-organic NSs, called nanocomposites or nanohybrids, are the result of their outstanding properties. Firstly, the multicomponent nature of nanocomposites produces specific physicochemical properties, but also the synergic effect confers unique properties to the whole system. Additionally, nanocomposites can be functionalized to selectively deliver drugs at specific cells, thus improving therapeutic efficacy. Surface functionalization, one-pot synthesis, and wrapping are among the main strategies for the construction of nanohybrids [37].

Surface functionalization is a powerful tool to provide nanohybrids with biocompatible hydrophilic surfaces and functional groups capable of interacting with biomolecules for targeting or delivery. Otherwise, one-pot synthesis offers a simple and efficient approach in which one of the components is formed directly during one step reaction in the presence of the other component. Wrapping means the encapsulation of one of the components with the other through noncovalent interactions. There are a variety of nanocomposites reported in the literature for which different materials combined into different architectures have been used [38,39].

**Figure 1:**
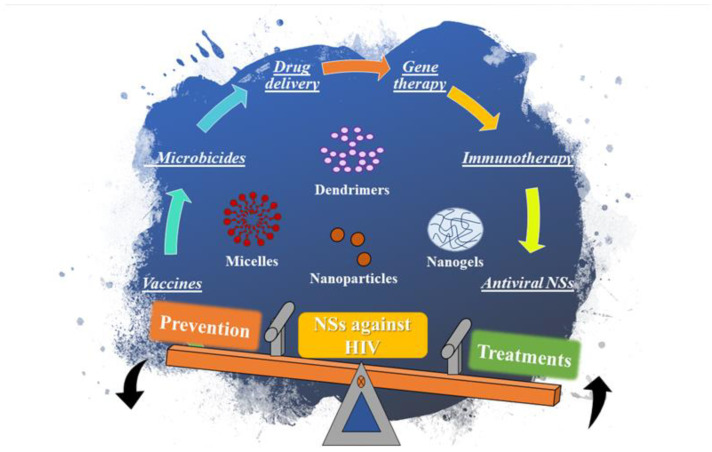
Scheme of the different approaches for prevention and treatment of HIV infections and several NSs described in this review.

After this overview of characteristics and advantages of different types of nanosized structures used for prevention and treatment of HIV infection, we describe the most recent and outstanding developments in NSs associated with prevention and treatment alternatives for HIV infection. Firstly, approaches for preventing HIV infection are addressed subclassing into vaccinology and topical microbicides. Then, we describe several NSs proved to be efficient for HIV/AIDS treatment such as for drug delivery, as the therapeutic agent, for immunotherapy, and gene therapy. The content of this review is schematically described in Figure 1.

## 2. Nanotechnology Approaches for Prevention of HIV Infection

Preventive strategies are the most effective actions to fight global infections. Vaccines have been successful at controlling other major infectious diseases such as measles, mumps, rubella, and polio, with smallpox completely eradicated [40]. In the absence of a preventive vaccine for HIV/AIDS, enormous efforts are made by researchers worldwide to achieve an effective formulation that passes the clinical trials.

Since sexual transmission is the major route of infection of HIV, another preventive strategy is the development of effective topical pre-exposure prophylaxis such as microbicides, which can be defined as medical products intended to be administered into the vagina and/or rectum in order to avoid early steps of viral transmission upon sexual intercourse [41]. The principle for dealing with new HIV infections relies on the inhibition of the virus at the mucosal level by one or several compounds, which have specific antiviral activity [41]. Despite the progress made in microbicide technology, the world is still awaiting approval of the first microbicide product, indicating the need for more research and development to design better systems.

### 2.1. Vaccines

As stated by Prof. Burton of the Scripps Research Institute in La Jolla, California, in the US, “of any pathogen, HIV provides perhaps the greatest challenge to successful vaccine development” [42]. An effective vaccine will contribute to elimination of HIV infection worldwide, however, candidate vaccines evaluated to date have failed to demonstrate efficacy [43]. A vaccine is an invention that certainly involves an empirical trial-and-error step that inherently differs from a rational design approach; in the case of HIV vaccines, the so-called rational design approach failed because the immunogenicity and antigenicity were confused [44]. The major barriers for HIV vaccine development are the failure to produce adequate vaccine immunogens and the inability of conventional delivery approaches to produce the needed immune response [45]. Therefore, significant efforts are still made to generate an efficacious vaccine for the prevention of HIV infection. In this context, NSs have exhibited outstanding properties as carriers for the improvement of solubility and pharmacokinetics of vaccine agents such as nucleic acids and therapeutic proteins [46]

A promising approach for developing HIV vaccines is the use of NPs as delivery agents for HIV antigenic peptides. As an example, nanovaccine formulation for HIV prevention was prepared by using chitosan/dextran sulfate NPs with the peptide antigen entrapped by ionic interactions [47]. Dacoba et al. engineered different polysaccharide NPs loaded with an HIV peptide antigen candidate, which is a sequence around the protease cleavage site 5 (PCS5) [48,49]. To form the NPs, PCS5 was first conjugated to two different polysaccharides (chitosan and hyaluronic acid) through either a stable or a cleavable bond and then, associated with an oppositely charged polymer (dextran sulfate and chitosan) and an immunomodulatory molecule, polyinosinic: polycytidylic acid (poly(I:C)). The results showed that different factors such as the attachment of the antigen (ionic interactions, and cleavable or noncleavable conjugations), the presence of immunomodulatory molecules such as poly(I:C), or the nature of the polysaccharides could importantly influence the type of elicited immune response. Regarding the delivery agents for HIV antigenic peptides, Martín-Moreno et al. used cationic nanocompounds, G4-70/30 dendrimer and the β-cyclodextrin derivative AMC6 to introduce HIV-1 peptides into human dendritic cells (DCs) [50]. Afterwards, the authors studied their maturation that makes them HIV-1-specific antigen-presenting cells to generate a T cell response when introduced back to the patient. Recently, HIV envelope glycoprotein (Env) was incorporated into different lipid assemblies. Micelles and nanodiscs with various lipid compositions were used. The authors used this methodology for studying Env in membranous environments, but it can also be adapted for vaccine engineering [51].

An immune-active nanovaccine delivery system to target DCs was designed using inulin acetate, which is a novel immune-active polymeric material (InAc-NPs) that targeted the TLR4 signaling on DCs for their activation and maturation [52]. Safety of this nanovaccine was demonstrated by their quick clearance from the injection site and the absence of skin toxicity. Additionally, in vivo InAc-NPs generated efficient humoral responses, demonstrating great potential in cancer immunotherapy and against various infectious diseases such as HIV.

An hybrid delivery system based on polyethylene glycol-graft-polyethylenimine (PEG-g-PEI)/DNA polyplexes formulated into poly(lactic-co-glycolic acid) (PLGA) microspheres was evaluated for DNA vaccine delivery [53]. Intramuscular injection of this DNA vaccine delivery system induces immune responses at a low dose of DNA in big animals such as guinea pigs and rhesus macaques. Therefore, this technology holds promise for use in human beings.

Table 1 summarizes the above examples and other recent developments for HIV vaccines.

### 2.2. Topical Microbicides

Microbicides are defined as topical prophylactic agents in the form of gels, creams, foams, impregnated sponges, suppositories, intravaginal rings, or films for self-administration into the vagina or rectum before intercourse to protect against HIV and other sexually-transmitted pathogens such as genital herpes, gonorrhea, and chlamydia [56]. In a general way, the chemical/physical action of these formulations protects the uninfected person, male or female, from infectious agents that might be present in the genital secretions of his/her sexual partner. One of the ways that sexual transmission of the virus can occur is through cervicovaginal or colorectal mucous membranes of receptive individuals upon contact with semen containing HIV.

**Figure 2:**
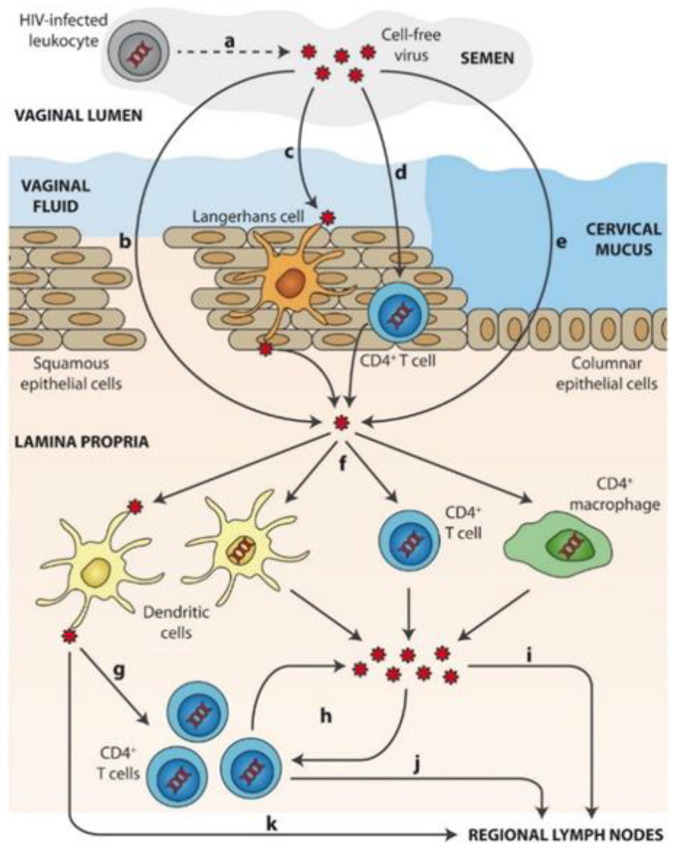
Mechanisms of sexual transmission of cell-free HIV through the cervicovaginal route. Reprinted with permission from [58].

Mechanisms of sexual transmission of cell-free HIV through the cervicovaginal route is schematized in Figure 2 [57,58]. After ejaculation, HIV present in semen or (a) produced by HIV-infected leukocytes from a donor requires first crossing the mucosal fluids. Then, viral particles can overcome the epithelial barrier by: (b) direct access to the lamina propria across gaps in the epithelium, (c) capture and transepithelial transport of virions by Langerhans cells, (d) partial penetration of the epithelium and infection of intraepithelial CD4+ T cells (or other leukocytes) that then migrate to the lamina propria, or (e) epithelial crossing through intercellular spaces or by transcytosis. Once in the lamina propria, (f) HIV particles can productively infect target cells such as macrophages, CD4+ T cells, or DCs. (g) DCs can also mediate trans-infection of other target cells, namely CD4+ T cells. Following initial infection of target cells, (h) local viral amplification occurs mainly in CD4+ T cells prior to migration of (i) free virus and/or (j) infected cells to regional lymph nodes. (k) HIV transfer to lymph nodes may also be mediated by non-productively infected DCs trans.

“Microbicide” is the most used term in literature even though there are some topical prevention strategies that do not kill microorganisms but do prevent their transmission. In this text, we refer to all these strategies as microbicides.

There are different topical prevention strategies currently under development that consist of agents that inactivate HIV directly: detergents and agents that modify pH, those that target viral replication or viral entry, and those which target host-cell structures [59].

First, microbicides must display preventive activity against HIV infection for several hours over a broad pH range, though this is not the only requirement that they should satisfy. In addition, they should guarantee successful application, distribution, and retention of the agent where it is needed. Therefore, viscosity and other physical characteristics should be optimized to ensure the most favorable antiviral activity, good coverage of the mucosa surface, sufficient tissue penetration if necessary, and a product that is as undetectable as possible [59]. Longer-acting tolerated agents are attractive because they might allow less-frequent administration [59]. Furthermore, microbicides have to demonstrate long-term safety without causing adverse effects, damage to mucosal integrity, inflammation or immunogenicity, or disturbing the normal vaginal flora.

In illustrating the most recent and relevant examples of research works involving NSs for the development of microbicides, it is important to mention the contribution of dendrimers. Considering their impact, VivaGel^®^ was the first dendrimer-based drug proposed as a new drug. VivaGel^®^ has potent virucidal activity against HIV-1 but toxicity studies in tissue explants, in vitro, animal, and non-human primate models show the relative safety of this product. Consequently, the development of VivaGel^®^ as a microbicide has been discontinued following safety issues in clinical trials [41]. Meanwhile, other candidates have been intensively investigated.

Extensive research has shown that polyanionic carbosilane dendrimers possess considerable anti-HIV-1 and anti-HIV-2 activity, namely due to their ability to bind to gp120 and CD4 and interfere with their interaction. Among different carbosilane dendrimers that have been studied, G2-S16 has emerged as one of the most promising candidates. In addition to its unquestionable microbicidal effect, it has demonstrated in vivo biocompatibility. Recent studies showed that G2-S16 dendrimer does not cause irritation or inflammation in the vaginal epithelium and does not alter the natural immunity of the vagina, which strongly supports the biosafety of this dendrimer for vaginal application to control viral transmission [60,61,62].

Moreover, polyanionic carbosilane dendrimers present synergistic effects when associated with antiretroviral (ARV) drugs. Regarding this, Sepúlveda–Crespo et al. studied the triple combination of anionic carbosilane dendrimers (G2-STE16, G2-S24P, and G2-S16) with tenofovir, maraviroc, or both against HIV-1 infection [63]. Combinations showed a greater broad-spectrum anti-HIV-1 activity than the single-drug, preserved this activity in acid environment or seminal fluid, and demonstrated strong synergistic interactions at high inhibitory concentrations. Finally, the treatment of vaginal epithelium in vivo (female BALB/c mice) showed no irritation. This result is a consequence of combining lower doses of different compounds that act synergistically, which leads to minimize systemic exposure and toxic side effects.

The development of prophylactic strategies with dual microbicide and contraceptive activity is also interesting. Platycodin D (PD), a promising contraceptive, was combined with G1-S2 or G2-S16 dendrimers to develop a prophylactic strategy with dual activity [64]. The results show that PD does not affect the antiviral activity of the dendrimers and they do not affect the spermicide activity of PD. The spermicide effect over human semen was achieved in less than 30 s.

Recently, anionic poly(alkylideneamine) dendrimers with carboxylate or sulfonate terminal groups were tested as microbicide agents against HIV-1 infection [65]. Dendrimers with eight carboxylate or sulfonate terminal groups (G1C and G1S dendrimers) showed important antiviral activity against infection both at acidic and basic pH values and, long term chemical stability in solid state and aqueous solution. In vivo assays using BALB/c mice revealed that G1C and G1S dendrimers did not cause noticeable irritation or inflammation in the vaginal epithelium.

Unlike dendrimers, there is little published data on nanogels as microbicidal agents. As example, poly(N-vinylcaprolactam) NGs demonstrated an inhibitory effect against HIV-1 infection by themselves [66].

Sánchez–López et al. prepared suitable delivery nanocarriers for releasing HIV-1 fusion inhibitor peptide in vaginal mucosa: polymeric NPs of PLGA and lipid large unilamellar vesicles loaded with the inhibitor peptide [67]. The authors comparatively studied both systems and found high entrapment efficiency of the inhibitor peptide in lipid vesicles, which was understood because of the hydrophobic nature of the peptide. PLGA NPs demonstrated an in vitro drug release similar to the free peptide whereas lipid vesicles demonstrated favorably prolonged release. Besides, none of the NSs were able to permeate across the vaginal tissue, thus probably avoiding adverse systemic effects in vivo. Lipid vesicles can deliver a sustained inhibitor peptide concentration in the vaginal tissue enhancing peptide penetration. Based on the results, lipid vesicles are a suitable formulation as a microbicide against HIV infection.

Bictegravir (BIC), a newly FDA-approved integrase strand transfer inhibitor, has proven efficacious in treating HIV-1. Recently, Mandal et al. investigated its prophylaxis effect [68]. PLGA-loaded BIC NPs demonstrated BIC therapeutic selectivity, intra-cellular delivery, retention, and sustained drug-release potency; improved BIC cytotoxicity; and enhanced HIV-1 protection compared to BIC in solution. Finally, polymeric protection increases the tolerability index of BIC compared to BIC solution. Furthermore, PLGA NPs loaded with two potent ARVs, griffithsin (GRFT) and dapivirine (DPV), were also evaluated as a potential long-acting microbicide product [69]. These drugs have different targets: the fusion and reverse transcription steps of HIV replication. Both were successfully encapsulated, GRFT (45% of initially added) and DPV (70%), and showed a biphasic release with initial burst phase followed by a sustained release phase. The combination of drugs in both unformulated and encapsulated in NPs showed strong synergistic drug activity. These findings showed that the co-delivery of GRFT and DPV promises to behave as a highly potent microbicide.

Table 2 summarizes some examples for HIV microbicides.

Recent work has studied a multilayered nanoparticle-electrospun fiber (NP-EF) composite for sustained-release of GRFT [75]. pH-responsive and surface-modified GRFT fibers provided in vitro long-term dual protection against herpes simplex virus type 2 (HSV-2) and HIV-1 infections. Composites were fabricated from polycaprolactone (PCL) fibers surrounding polyethylene oxide (PEO) fibers that incorporated methoxy poly(ethylene glycol)-b-poly(lactide-co-glycolide) (mPEG-PLGA) GRFT NPs. High loading of GRFT NPs and sustained release of GRFT during a 90-day period were achieved. Both NPs and NP-EF composites inhibited HIV-1 infection in vitro, and moreover, these vehicles demonstrated protection against a lethal dose of HSV-2 infection in a murine model. The data indicate the preliminary safety and biocompatibility of these delivery platforms.

In contrast to formulations for vagina, there is much less information about rectal anti-HIV microbicides. José das Neves and col. reported experimental work about the in vitro and in vivo performance of PLGA-based NPs as carriers for the model drug efavirenz (EFV) for intrarectal administration [76]. In particular, the effect of non-covalent PEG coating of PLGA NPs (PEG-PLGA NPs) on the pharmacokinetics of EFV following rectal administration to mice was assessed. Both drug-loaded PLGA-NPs and PEG-PLGA NPs improved the colorectal availability of EFV after rectal administration as compared to free drug. Nevertheless, prolonged drug residence at the lower colon was observed at higher concentrations when PEG modification was used. Thereby, this work gives evidence of the usefulness of mucus-diffusive nanocarriers in engineering effective and safe rectal microbicides.

## 3. Nanotechnology Approaches for HIV/AIDS Treatment

At present, there is no cure for HIV/AIDS, and in the absence of treatment, HIV infection causes death in a period between 5–10 years [77]. In this context, lifetime treatments have been used to improve expectancy and quality of lives for HIV-infected patients. The current treatment modality for HIV/AIDS is antiretroviral therapy (ART) or highly active antiretroviral therapy (HAART), where a combination of three or more different classes of ARV drugs are administered. Taking into account the HIV replication life cycle and the known molecular targets of HIV, a significant number of highly potent orally administered ARV drugs have been approved and combined in HAART to suppress HIV replication and thus, limit disease progression.

HAART treatments have achieved remarkable success in the fight against HIV/AIDS, however, there are still various challenges remaining. As the treatment requires medication daily for a lifetime, one of the major reasons for its failure is due to poor patient compliance [40]. On the other hand, the selection of the combined drugs used in HAART in not an easy task. The aim is to choose different classes of drugs that work by different mechanisms and, therefore, the selection depends upon various factors like drug properties, drug cost, drug resistance status, and patient characteristics [40,77]. Another important drawback of this therapy is the short residence time of ARV drug that leads to a decrease in the concentration of drug at sites of viral reservoirs like lymphatic system, macrophages, lymphocytes, central nervous system, and lungs. Consequently, higher doses of drugs are required over a long period of time. Long-term therapy can generate resistance in HIV strains. Thereby, drug resistance can cause failure of ARV treatment by ineffective viral suppression, especially in viral reservoir sites. Finally, inherent drug toxicity, adverse drug effects, and drug–drug interaction between ARV drugs and other drugs are other issues to consider in HIV treatment.

So far there are no efficient therapies against HIV infection, only two cases have been reported in which the virus has been completely eradicated [78,79]. Under current treatment, complete eradication of the virus from the body is not possible because, as it was mentioned before, HIV can establish viral reservoirs to maintain persistent infection. Patients receiving a combination of anti-HIV drugs have undetectable plasma viral loads, but they still carry the virus in reservoir sites [80]. In this context, there is a great need to develop targeted delivery of ARV drugs to latent reservoirs to eradicate the virus from reservoirs.

Although the main approach that involves NSs in the therapy of HIV infection is their use as drug nanocarriers, other strategies have also been developed including NSs with antiviral ability by themselves or NSs to improve immunotherapy and gene therapy. In this section, we list some of the most recent examples. Furthermore, this new generation of medicines needs to be of low toxicity and lower-dosage modalities that provide more sustained dosing coverage and effectively avoid the need for lifetime treatments. Different types of nanotechnology approaches for HIV/AIDS treatment classified by therapy approach and NSs type are shown in Table 3.

### 3.1. Drug Delivery Nanosystems

**Figure 3:**
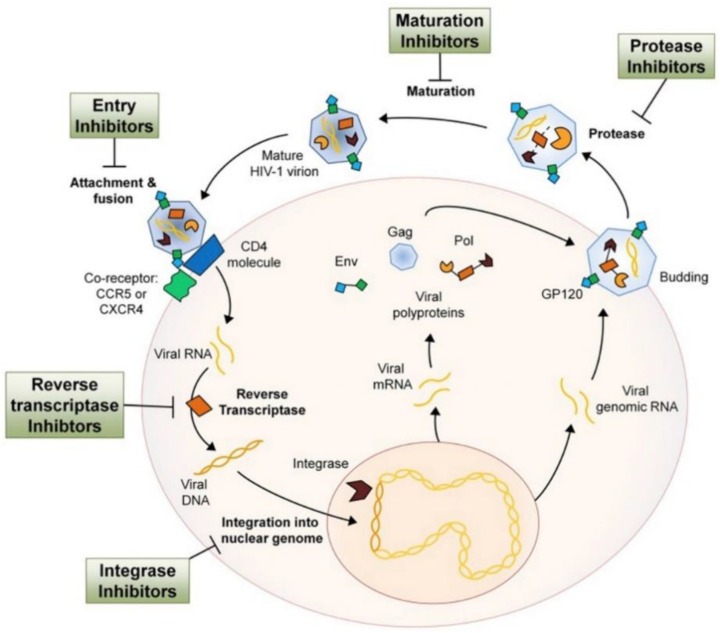
Schematic description of the mechanism of currently available ARV drugs against HIV. Reprinted with permission from [96].

ARV drugs are usually classified by the phase of the retrovirus life cycle that the drug inhibits. These phases and the different mechanisms of action of ARV (entry, reverse-transcriptase, integrase, protease, and maturation inhibitors) are schematized in Figure 3.

In order to overcome some of the major disadvantages of ARVs in HIV treatment, it is not surprising that NSs have gained significant attention as potential solutions to address these adversities. Their nanometric size in combination with unique properties such as the capability to target specific cells by passive or active target, load ARV drugs with poor solubility or stability, maintain drug concentration levels in site of action, and improve circulation time, among others [97,98] makes these type of systems promising in HIV/AIDS treatment. Their application is mainly focused on producing modifications in the bioavailability and pharmacokinetics of ARVs, which leads to an improved and enhanced antiviral effect [99,100,101].

A wide range of NSs are being studied as possible candidates for ARVs carriers. Among the most used systems, liposomes, niosomes, polymers, solid-lipid NPs, nanohybrid, and dendrimers are the most relevant ones [41,46,102,103]. In this section, we discuss ART common problems and how NSs are being employed to treat HIV infection and their strategies to enhance treatment efficiency, biocompatibility, and bioavailability, and reduce toxicity as well as drug interactions with undesired targets.

ARV-related issues such as low solubility and stability, non-favorable interactions, and toxicity are currently being solved by employing nanocarriers with tunable properties, high loading ARV capacity, sustained and selective release of cargo molecules, and attached target molecules [104,105,106]. For example, Chiapetta et al. designed and synthetized mixed polymeric micelles of poloxamer/poloxamines and encapsulated efavirenz with a sharp increase in encapsulation efficiency, up to 8000-fold increase [81]. Additionally, Ramana et al. reported a chitosan-based NSs for saquinavir delivery, a low bioavailable drug. Chitosan is a positively charged biopolymer, which presents enhanced biocompatibility and immunogenicity, as well as anti-microbial properties. In addition, a positively charged surface provides a superior targeting efficiency to negatively charged cells and favors proteins deposition by enhancing NS uptake by macrophages, where drug release occurs under acidic endosome conditions. Chitosan-based NSs exhibited high saquinavir loading capacity, up to 80% with an average size of 210 nm and great cell targeting efficiency over 90%. These saquinavir-loaded NSs exhibited superior potency compared with free drug, resulting as promising candidates for HIV delivery system [82].

Alongside known ARV problems, a major drawback to be solved is the impossibility for these drugs to cross the blood brain barrier (BBB), which should be taken into account since it is not a minor adversity. BBB is a highly selective and regulated membrane that controls the movement of molecules, ions, or cells into the central nervous system (CNS), greatly hindering ARVs entrance to brain. Since HIV is capable to establish viral reservoirs in the brain, lymphoid organs, and nodes or macrophages [107,108,109,110], it results in being impossible to completely erase the virus. Thus, delivery through BBB remains a difficult task to carry out. Nano-delivery systems arise as an alternative strategy to achieve ARV delivery through the BBB [111,112]. Their controllable size along with large surface-to-volume ratio, and tunable properties [80,113,114,115] provide key desired aspects that can easily support ARV transport to the brain, where principal HIV reservoirs are located [116,117]. Belgamwar et al. synthetized intranasal (IN) delivery NSs composed of cyclodextrin-grafted chitosan (CD-g-chitosan) with loaded efavirenz to deliver through the BBB. NSs for IN delivery presented enhanced bioavailability of the drug, since it offered drug protection against biological and chemical degradation and against rapid elimination of the hepatic system or efflux proteins. Developed CD-g-chitosan NSs improved efavirenz solubility and mucosal adhesivity while maintaining chitosan natural properties. IN nanoformulations were employed in order to enhance efavirenz delivery to CNS. These NSs with an average size of 200 nm, presented five times higher permeability and enhanced bioavailability up to 12 times free efavirenz; high drug targeting percentage has also been found [83]. Another desirable property of NSs for delivery is the capacity to encapsulate different drugs in the same particulate material and release them independently. In this regard, Choi et al. developed lipid-indinavir NSs by ultrasonication and studied the encapsulation and release of ^3^H-phosphonylmethoxypropyl-adenine (PMPA), two of the most used ARVs for HIV treatment. The lipidic NSs presented enhanced release of encapsulated PMPA when trapped in a indinavir-lipid membrane, but pH-mediated release of indinavir affected PMPA release [84]. The design and development of multi-drug delivery systems remains a challenge because achieving independent drug release is still a difficult property to control.

The major disadvantage of HAART as inhibitory therapy is the inability to eliminate the virus, thus patients need to take medication periodically throughout their lives. If a patient discontinues HAART treatment (due to drug toxicity or inconvenience in medication uptake) or drug resistance is developed, HIV viral load could rise in response to treatment failure. Long-acting (LA) ARV formulations are desired systems to sustain drug concentrations for longer periods, reducing the intake time between HAART doses to weeks or even months [118,119]. The successful design of LA systems should bring about an improvement in the quality of patient life, as well as a better efficiency in the treatment. Lamivudine is a commonly employed HAART drug that exhibits some limitations like low protein bound (<36%), half-life time of 6 h, and rapid renal clearance [120]. In order to improve lamivudine properties, Guo et al. developed a novel nanoformulation of a hydrophobic prodrug, a myristoylated/lamivudine conjugate wrapped in a surface-decorated poloxamer 407, with potentially higher antiretroviral potency compared to free drugs. NSs surface was modified with folic acid (FA), which is a well-known molecule to target macrophages, to enhance cellular target and cellular drug uptake. The authors studied lamivudine levels found at different times in mice and compared FA-targeted NSs with non-targeted NSs and free pro-drug. They found the best result with FA-targeted NSs with high cellular uptake in the first hours and a sustained release of lamivudine up to 2 weeks. Non-targeted systems presented significant lower cellular uptake and rapid release within a few hours of administration. In addition, drug levels found in different mice organs are higher for FA-targeted NSs. Thereby, the results showed that a novel lamivudine prodrug was successfully synthetized and incorporated into nanoformulations with reduced toxicity, improving half-life and a sustained release of drug in interest site [85].

As it was stated above, the primary goal of LA systems is to maintain enough drug level to control HIV viral load and prevent drug resistance development. In addition, they tend to solve problems associated with missed doses. In recent years, systems with the capacity to sustain drug release up to years have emerged as ultra-long-acting systems [80,85,121,122,123,124,125].

A complementary strategy to HAART is the reactivation of latent viral reservoirs in cells by molecules known as latency reversing agents (LRAs). The general idea is to reactivate HIV virus whilst maintaining HAART treatment, thus viral load could be minimized, and new infections and the formation of newer viral reservoirs could be prevented. Nonetheless, this strategy lacks an efficient way to minimize viral reservoirs [126,127]. On the other hand, it has been studied that combinations of different LRAs would reduce dose levels and produce a synergistic effect among combined LRAs with reduced toxicity. Considering this, Relaño–Rodriguez et al. studied the role of anionic carbosilane dendrimers in combination with known LRA, bryostatin, romidepsin, and panobinostat, to be used in LRA combined therapy. The combination of LRA with different generations of dendrimers led to an increase in the replication of GFP cell lines, suggesting a new approach for combination therapy [86].

### 3.2. Nanosystems as Therapeutic Agents

Some newfangled NSs have presented great efficacy against virus infection by themselves. Although in most cases the mechanism is not noticeably clear, NSs can act over the fusion or entry of the virus target cell and this leads to inhibition of viral activity and viral cytotoxicity. Chen et al. offered a broad review about antiviral efficacy and the mechanism of these NSs applied to different kinds of viruses [128]. Specifically, different NSs have been tested in vitro for the HIV based on gold [72,87,129], silver [88,130], and silica nanoparticles [89]; liposomes [131]; and quantum dots [90,91].

Vijayakumar et al. obtained gold nanoparticles (AuNPs) by a modification of the Turkevich method and stabilized with PEG. They tested their cytotoxicity and antiviral activity against HIV-1 under in vitro conditions. In the cell-based fusion assay, thiocarboxanilide non-nucleoside reverse transcriptase inhibitor ARV drug (UC-781) was used as control. Although the mechanism of AuNPs against HIV-1 is not clear, these AuNPs presented excellent activity as a virucidal agent or viral entry inhibitor [87]. Likewise, Peña–Gonzalez et al. studied the inhibitory action of dendronized AuNPs and dendrons against HIV-1 infection. They synthesized a series of AuNPs conjugated with anionic dendrons with sulfonate functions and one thiol moiety at the focal point. Their results showed that dendrons by themselves presented a lower efficiency than dendronized AuNPs; toxicity was decreased; and antiviral activity was increased with respect to pioneering dendron compounds [72]. Also, NSs of silver were tested; Etemadzade et al. developed silver nanorods conjugated with sodium 2-mercaptoethane sulfonate (Ag-MES) and evaluated their potential antiviral activity against HIV and herpes simplex virus type 1 (HSV-1) viruses in human cervical cancer HeLa cells. They stated that metal NPs conjugated with MES can block adhesion between virus and the host cell. Their study showed Ag-MES almost inhibited entire viral replication. However, HIV showed more sensitivity than HSV-1 [88].

Silica nanoparticles both porous and non-porous are biodegradable. In aqueous media they hydrolyzed in non-toxic silicic acid, which is cleared from kidney through urine [132,133]. Along with other properties, this biocompatibility has made these NPs widely investigated in different biomedical applications. Osminkina et al. used mesoporous silica nanoparticles (MSNs) as scavengers of HIV and respiratory syncytial virus (RSV). They obtained MSNs with 5–50 nm of size through a mechanical method, studied the cytotoxicity of the MSNs in various cell lines, and evaluated the virucidal activity in HIV (HIV-1BRU strain) or RSV (Long strain). They found that 50 % inhibition was reached when the MSNs concentration were 0.1 and 0.005 mg/mL for HIV and RSV, respectively, which was significantly lower than the cytotoxic concentration. The obtained results are assigned to the unique porous structure of this type of NSs. MSNs can bind to viral proteins through a non-specific interaction, which can effectively reduce the infectious virus strains and change the virus attack behavior [89].

In this same sense, quantum dots (QDs) have been studied as therapeutic agents. QDs are semiconductor nanocrystals with unique optical and electronics properties dependent on their size. Due to their unequaled luminescent properties, they are widely used as labeling in image tracking [128]. Recently, various harmless methods of antiviral treatment using QDs have been reported. Aung et al. proposed amino phenylboronic acid-modified carbon dots (APBA-CDs) that inhibit HIV-1 entry processes. These NSs were synthesized through a simple pyrolysis process, decarboxylation, and self-assembly reactions. In this way, they acquired a graphene-like structure on carbon dots that improves its biocompatibility and high stability properties. The data demonstrated that the modified carbon dots exhibited superior capabilities in terms of prohibiting HIV-1 entry into target cells by themselves. Furthermore, the authors demonstrated a synergistic extracellular viral blocking when combining the APBA-CDs with the Duviral drug [90]. Another example of QDs as a therapeutic agent was proposed by Iannazzo et al. They synthesized QDs from multi-walled carbon nanotubes (MWCNT) through prolonged acidic oxidation and exfoliation. Then, antiviral activity of the QDs was studied and compared with the one obtained by QDs conjugated with CHI499 and CDF119, which were poorly water-soluble non-nucleoside reverse transcriptase inhibitors. The obtained results showed that QDs present a highlight antiviral activity and these NSs improved the antiviral activity of the conjugated compounds since the solubility of these compounds notably increases when conjugated with the QDs [91].

### 3.3. Immunotherapy

The HIV genome, like any other retrovirus, is constituted by a single strand of RNA that is reverse transcribed into DNA through the reverse transcriptase enzyme and integrated with the host genome. Principally, the HIV attacks CD4+ T lymphocytes leading to their depletion and the consequent decrease of the innate immune response of the infected person [134]. The ART can control the HIV replication life cycle and prevent the developing AIDS in the patient. However, HIV has the ability to generate reservoirs in some tissues and it keeps it undetectable; this makes it more difficult to get an effective cure.

The eradication of the residual infection could be achieved through an immunological approach. This includes prevention, given by the development of vaccines, as well as the improvement of pre-existing therapies, through active targeting, as previously explained in this review. However, most of this research has not shown sufficient efficacy and, in some cases, safety issues have been presented. The immune approach that involves antibodies for viral clearance appears to be a safe promising therapeutic alternative against HIV infection. The clearance by immune cells consists of the transplantation of antibodies to the patient after ex vivo expansion and/or modification to act on infected cells. It aims to recognize and enhance HIV specific cytolytic T cell responses [135]. The antibodies can be either owned (autologous) or from a donor (allogenic). Additionally, they can be sorted out in either neutralizing antibodies (Nabs) or no neutralizing antibodies (Non-Nabs). The action of the first ones is directed at blocking infection by binding to the envelope of free viruses, while the other is toward recognition of the envelope during virus entry or on the surface of infected cells [136,137].

The antibody treatment has been shown to be effective, both in vitro and in vivo studies [138,139,140,141,142,143,144,145], in the clearance of the free virus, elimination of infected T cells, as well as reduction of proviral DNA [43]. In fact, advances in antibody engineering have made this approach the most clinically advanced [136,146,147,148].

In this sense, the conjugation of this immune approach with NSs can provide an improvement in the ability of antibodies to recognize and attack infected cells. For example, Sweeney et al. proposed a nano-immunoengineering approach to improve the cytotoxicity of natural killer (NK) cells as antiviral and antitumor agents. The NK cells are a type of lymphocyte, but they cannot be infected by HIV because they lack the required surface receptors allowing them to exert cytotoxic pressure without the risk of viral spread. In this study, the authors developed a PLGA NSs that co-encapsulated prostratin, a LRAs, and anti-CD25, a cell surface binding antibody found on target cells. In an in vitro model of latent HIV, NSs exhibited successfully released both active prostratin and anti-CD25, and with controllable release kinetics. Besides, NSs were able to increase NK cell cytotoxicity of the target cells [92]. In another study, Zhang et al. designed PLGA NPs coated with the plasma membranes of uninfected CD4+ T cells. They demonstrated the potential to neutralize a broad range of HIV-1 strains by inhibiting replication. Furthermore, these NSs were able to selectively bind to infected CD4+ T cells and macrophages, without having any effect on uninfected cells [93].

### 3.4. Gene Therapy

**Figure 4:**
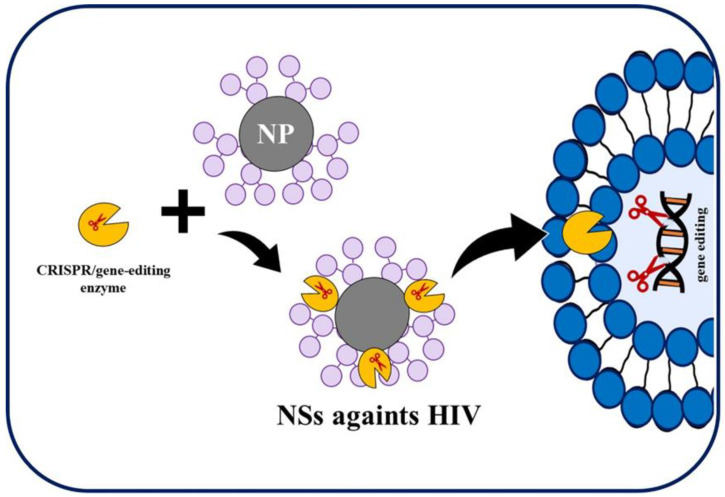
Indicative scheme of the gene editing approach.

This therapy is defined as the technique that allows to introduce corrective genetic material into cells as treatment of diseases; it uses genetic material, such as plasmids, nucleic acids (DNA and RNA), and oligonucleotides [149,150]. In addition, this therapy is also associated with the tool that allows genome editing or gene editing, which makes it possible to add, remove, or alter genetic material at locations in the genome, schematized in Figure 4. [151]. Specifically, nine genes are involved in the HIV-1 replicative cycle such as gag, pol, vif, vpr, tat, rev, vpu, env, and nef [152]. The targeting of small interfering RNA (siRNA) to them may silence their expression and control viral replication [153]. To apply this strategy, it is necessary to use gene delivery carriers. They can be viral and non-viral, but viruses and their modifications show considerable disadvantages due to their nature [154].

In the same way, gene-editing approach, commonly named CRISPR (clustered regularly interspaced short palindromic repeats), has presented a growing interest as an approach to eradicate HIV/AIDS [151,155,156,157,158]. Several enzymes have been studied to cut HIV provirus from the host genome such as homing endonucleases, evolved recombinases, zinc finger nucleases (ZFNs), transcription activator-like effector nucleases (TALENs), and CRISPR/Cas9 [43].

Each tool comes with its own benefits and limitations. Both siRNA and gene editing tools still have challenges in achieving an efficacy and safety cure for HIV. For example, these naked tools are easily degraded and exhibit low delivery in the cellular environment. However, some investigations suggest that the conjugation of these tools with NSs could improve their therapeutic action [94,95,154,159,160,161,162].

The study developed by Mobarakeh et al. is a clear example of the use of NSs to increase the therapeutic efficiency of these tools. They used modified chitosan NPs to introduce anti-HIV siRNA into different cell lines. The results demonstrated that NSs noticeably increased siRNA delivery efficiency with no significant cytotoxicity. In addition, NPs significantly reduced the RNA and protein expression of HIV-1 [94]. On the other hand, current CRISPR-Cas9 delivery techniques use electroporation to facilitate DNA entry into living cells, which is difficult to control and can generate cytotoxicity [137]. Shahbazi et al. showed how the use of AuNPs can mediate CRISPR-Cas9 components to target cells with higher efficiency and lower cytotoxicity [160]. Analogously, Kaushik et al. demonstrated that the use of magneto-electric NPs (MENPs) considerably increases the therapeutic action of Cas9/gRNA. They achieved for the first time magnetically-guided non-invasive delivery of Cas9/gRNA/MENPs, across the BBB to inhibit latent HIV-1 infection [95].

## 4. Concluding Remarks and Future Perspectives

During the last three decades, great improvements in the diagnosis, treatment, and prevention of HIV have been made. Cabotegravir and rilpivirine are two long-acting retroviral nanoformulations whose preclinical and clinical trials, respectively, have been successful. Their discovery demonstrates the benefits of this type of formulation on the nanoscale: greater potency, half-life, and sustained circulation time in plasma, giving a greater amount of the drug deposited in specific tissues. Since then, many efforts have been made in research focused on the goal of viral eradication and improvement of new drugs, NSs and their possible combinations. This includes a better understanding of the effects of charge, size, structure, and potential toxic side effects of NSs. In addition, as understanding of the many mechanisms of viral persistence increases, new approaches to interrupt and to target latency are moving towards clinical testing.

There is great hope in nanogel-drug conjugated systems that improve the efficacy of antiviral therapy. This is due not only to the aforementioned advantages, but because of their size, they can cross the BBB and act on the central nervous system, eradicating HIV infection in the brain.

Without a doubt, prevention is better than cure, therefore, the ultimate goal would be to provide an easy-to-use option against sexually-transmitted diseases for sexually active people. The use of effective microbicides, condoms, and eventually AIDS vaccines will give society a wider variety of protective technologies.

Finally, the cure for HIV that was once considered an unattainable goal is now a reality, and its eradication is an achievable short-term goal. The greatest hope lies in the use of antibodies and the development of vaccines against HIV.

## Figures and Tables

**Table 1:** Nanotechnology approaches for HIV vaccines.

Prevention Approach	Goal	Nanoformulation	References
Vaccines	Transport of HIV antigens to targeted immune cells	Chitosan/dextran sulfate NPs with HIV antigenic peptides	[48,49]
		G4-70/30 dendrimer and the β-cyclodextrin derivative AMC6 for peptide delivery	[50]
		Complex based on fourth- generation poly(amidoamine) dendrimers (G4-PAMAM) and peptide epitopes	[54]
		PLGA NPs with HIV antigenic peptide conjugated to an adjuvant	[55]
		Inulin acetate NPs with encapsulated antigen (ovalbumin)	[52]
	DNA vaccine delivery	(PEG-g-PEI)/DNA polyplexes formulated into PLGA microspheres	[53]

**Table 2:** Nanotechnology approaches for HIV microbicides.

*Topical Microbicides*	**Type of NSs**	**NSs or Nanoformulation**	**Mechanism/Description**	**References**
	Carbosilane dendrimers	Anionic carbosilane dendrimers: G2-S16 and others	Anti-HIV-1 and anti-HIV-2 activity due to their ability to bind to gp120 and CD4 and interfere with their interaction.	[60,61,62] [70,71]
		associated with nanoparticles, fatty acids		[72,73,74]
		associated with antiretroviral drugs, contraceptives		[63,64]
	Anionic poly(alkylideneamine) dendrimers with carboxylate or sulfonate terminal groups	G1C and G1S dendrimers	Antiviral activity against infection at acidic and basic pH values, long term chemical stability.	[65]
	Polymeric systems	Poly(N-vinylcaprolactam) NGs	Inhibitory effect against HIV-1 infection by themselves.	[66]
		PLGA NPS and lipid large unilamellar vesicles loaded with the inhibitor peptide	Release of HIV-1 fusion inhibitor peptide in vaginal mucosa.	[67]
		PLGA-loaded Bictegravir NPs	Bictegravir, an integrase strand transfer inhibitor, tested for prophylaxis.	[68]
		PLGA NPs loaded with antiretrovirals: griffithsin and dapivirine	The combination of drugs showed strong synergistic drug activity.	[69]
		PCL fibers surrounding PEO fibers that incorporated mPEG-PLGA NPs loaded with griffithsin	Sustained release of griffithsin during 90-day period were achieved.	[75]
		PLGA NPs as carriers for efavirenz	For intrarectal administration.	[76]

**Table 3:** Nanotechnology approaches for HIV/AIDS treatment.

Therapy Approach	NSs Class	Drug	Description	References
Drug delivery nanosystems	Mixed poloxamine/poloxamer polymeric micelles	Efavirenz	NSs greatly improved efavirenz solubility for oral administration. Drug-loaded mixed micelles also exhibited enhanced physical stability compared to pure drug.	[81]
	Polymeric NPs	Saquinavir	A Chitosan nanoformulation with saquinavir-enhanced bioavailability. In addition, poly cationic chitosan improved macrophages uptake by negatively charged proteins deposition on its surface.	[82]
	Cyclodextrin-polymeric NPs	Efavirenz	Efavirenz-loaded chitosan-cyclodextrin NSs for intranasal administration showed sustained drug release; greater permeability than free drug; high encapsulation; and enhanced CNS bioavailability.	[83]
	Lipidic NPs	Indinavir & PMPA	Lipid-indinavir particles with the capacity to encapsulate PMPA and calcein, and further deliver to lymph nodes and tissues.	[84]
	Poloxamer Conjugate	Lamivudine	A folic acid-modified poloxamer with entrapped lamivudine was synthetized as a long acting nanoformulation. These NSs presented improved macrophage uptake, drug bioavailability and pharmacokinetics of drug combined with slow release.	[85]
	Dendrimers	Bryostatin, Romidepsin & Panobinostat	Carbosilane dendrimers in combination with different LRA-produced viral reactivation in THP89GFP monocyte cell line as a new approach for HIV treatment.	[86]
*Nanosystems as therapeutic agents*	AuNPs conjugated with polyethylene glycol	No	These NSs presented excellent activity as a virucidal agent or viral entry inhibitor. Although, the mechanism of AuNPs against HIV-1 is not clear.	[87]
	AuNPs conjugated with anionic dendrons	No	Dendrons by themselves presented a lower efficiency than dendronized AuNPs; the results showed that the toxicity is decreased and antiviral activity is increased with respect to pioneering dendron compounds.	[72]
	Silver nanorods conjugated with sodium 2-mercaptoethane sulfonate (Ag-MES)	No	The NSs can block adhesion between the virus and the host cell. These NSs almost inhibited the entire viral replication of HIV and HSV-1 in human cervical cancer HeLa cells	[88]
	MSNs	No	The NSs can bind to viral proteins through a non-specific interaction, which can effectively reduce the infectious virus strains and change the virus attack behavior.	[89]
	Amino phenylboronic acid-modified carbon dots (APBA-CDs)	Duviral	The NSs avoid HIV-1 entry into target cells by themselves. Besides, the synergistic extracellular viral blocking when combining the APBA-CDs with the Duviral drug was demonstrated in MT-4/HIV-1 and MOLT-4 cells.	[90]
	QDs from multi-walled carbon nanotubes (MWCNT)	CHI499 and CDF119	The NSs exhibited a highlight antiviral activity for themselves, which improved the antiviral activity of the conjugated system since the solubility of these compounds notably increases when conjugated with the QDs.	[91]
*Immunotherapy*	PLGA NPs	Prostratin	The NSs encapsulated a LRA and anti-CD25, an antibody that binds to CD25 expressed on the surface of the target cells. In an in vitro model of latent HIV, the NSs exhibited successfull release of both. Moreover, the cytotoxicity of NK cells as antiviral or antitumor agents was enhanced.	[92]
	PLGA NPs coated with the plasma membranes of uninfected CD4+ T cells	No	The NSs presented potential to neutralize a broad range of HIV-1 strains by inhibiting replication and to induce cell death in macrophages and CD4+ T cells infected with HIV.	[93]
*Gene therapy*	Chitosan NPs modified with polyethylenimine and carboxymethyl dextran	No	The NSs noticeably increased siRNA delivery efficiency with no significant cytotoxicity. In addition, the NSs significantly reduced the RNA and protein expression of HIV-1 into two mammalian cell lines.	[94]
	Cas9/gRNA bound with magneto-electric NPs (MENPs)	No	The outcomes suggested that the NSs reduced HIV-1 infection expression significantly in comparison to unbound Cas9/gRNA. They achieved for the first time magnetically guided non-invasive delivery of Cas9/gRNA/MENPs, across the blood brain barrier.	[95]
